# Pathogenicity and intestinal barrier disruptive ability of *Malassezia furfur* in an alternative model host *Caenorhabditis elegans* is partially alleviated by *Lacticaseibacillus rhamnosus*

**DOI:** 10.1093/femsmc/xtag049

**Published:** 2026-09-08

**Authors:** Chiho Kishida, Kanamu Chikuba, Chinatsu Yamamura, Himari Kanatani, Ayano Tsuru, Satoka Takabayashi, Xueyang Wu, Misaki Okahata, Yoshihiko Tanimoto, Eriko Kage-Nakadai

**Affiliations:** Graduate School of Human Life and Ecology, Osaka Metropolitan University, Osaka 558-8585, Japan; Graduate School of Biostudies, Kyoto University, Kyoto 606-8501, Japan; Graduate School of Human Life and Ecology, Osaka Metropolitan University, Osaka 558-8585, Japan; Graduate School of Biostudies, Kyoto University, Kyoto 606-8501, Japan; Graduate School of Human Life Science, Osaka City University, Osaka 558-8585, Japan; Department of Food and Nutrition, Faculty of Human Life Science, Osaka City University, Osaka 558-8585, Japan; Graduate School of Human Life Science, Osaka City University, Osaka 558-8585, Japan; Graduate School of Biostudies, Kyoto University, Kyoto 606-8501, Japan; Graduate School of Pharmaceutical Sciences, Kyoto University, Kyoto 606-8501, Japan; Institute for Life and Medical Sciences, Kyoto University, Kyoto 606-8507, Japan; Graduate School of Human Life and Ecology, Osaka Metropolitan University, Osaka 558-8585, Japan; Graduate School of Biostudies, Kyoto University, Kyoto 606-8501, Japan; Graduate School of Pharmaceutical Sciences, Kyoto University, Kyoto 606-8501, Japan; Institute for Life and Medical Sciences, Kyoto University, Kyoto 606-8507, Japan; Graduate School of Human Life and Ecology, Osaka Metropolitan University, Osaka 558-8585, Japan; Graduate School of Biostudies, Kyoto University, Kyoto 606-8501, Japan; Graduate School of Pharmaceutical Sciences, Kyoto University, Kyoto 606-8501, Japan; Institute for Life and Medical Sciences, Kyoto University, Kyoto 606-8507, Japan

**Keywords:** *Malassezia furfur*, Pathogenicity, *Caenorhabditis elegans*, *Lacticaseibacillus rhamnosus*

## Abstract

*Malassezia furfur* is fungi associated with various diseases; however, the mechanisms underlying its pathogenicity and the relationship between probiotics and fungi remain largely unknown. In the present study, *Caenorhabditis elegans* was used as the model host to evaluate *M. furfur* pathogenicity. Additionally, effects of lactic acid bacteria against *M. furfur* pathogenicity were evaluated. Compared to *Escherichia coli* OP50 (OP, control), both live and heat-killed *M. furfur* reduced the lifespan and body size of *C. elegans*, although heat-killed *M. furfur* was less effective than live *M. furfur* in lifespan shortening. Furthermore, unlike heat-killed *M. furfur*, live *M. furfur* disrupted the nematode intestinal barrier. Loss-of-function mutants of *nsy-1* and *sek-1*, which encode components of the MAPK signaling pathway, were susceptible to *M. furfur*, suggesting their involvement in the defense against *M. furfur* infection. Expression of genes involved in host defense and of those coding for C-type lectin domain-containing proteins and antimicrobial peptides was upregulated in *M. furfur*-infected *C. elegans. Lacticaseibacillus rhamnosus* (LR) significantly ameliorated lifespan shortening and body size reduction in *M. furfur*-infected *C. elegans* and protected against intestinal barrier disruption, suggesting that LR protects nematodes from *M. furfur* virulence. This study highlights *M. furfur* pathogenicity and intestinal barrier disruptive ability in *C. elegans* and suggests that the *M. furfur* virulence is partially attenuated by LR.

## Teaser

Malassezia furfur exhibited pathogenicity toward the model host C. elegans, and this pathogenicity was attenuated by feeding lactic acid bacteria.

## Introduction

*Malassezia* is a dimorphic fungus found in the human skin microbiota and dominates more than half of all the fungal species on the skin (Gao et al. 2010). *Malassezia* requires lipids for growth and is predominantly found in lipid-rich areas of the human body, such as the scalp and trunk (Prohic et al. 2016). The fungus is present on the skin under normal conditions and is associated with various cutaneous and systemic diseases, including pityriasis versicolor, folliculitis, seborrheic dermatitis, as well as with sepsis related to deep venous catheters in infants (Borgers et al. 1987, Elewski 1990, Piérard-Franchimont et al. 1995, Ashbee et al. 2002). Although the exact mechanisms underlying these diseases are not fully understood, hyphal formation in patients with pityriasis versicolor and upregulation of lipases and metabolic product (such as indole) in patients with seborrheic dermatitis has been reported (Prohic and Ozegovic 2007, Gaitanis George et al. 2008, Patĩo-Uzcátegui et al. 2011). Furthermore, *Malassezia* has been found not only on the skin but also in the gut of patients with inflammatory bowel disease, and mucosal lavage samples from patients with Crohn’s disease had increased levels of *Malassezia* compared to healthy controls (Limon et al. 2019). Nevertheless, interactions between *Malassezia* and its host remain ambiguous and studies to determine its potential as a contaminant are warranted owing to *Malassezia* being a cutaneous microflora (Abdillah and Ranque 2021).

Currently, the genus *Malassezia* comprises 18 species (Lorch et al. 2018). Among these, *Malassezia furfur* has been implicated in various skin diseases, including pityriasis versicolor and seborrheic dermatitis (Gaitanis Georgios et al. 2012). The genus is also the most frequently identified in bloodstream infections, which occur via catheters in immunocompromised patients and neonates (Rhimi et al. 2020). Several indoles induced by *M. furfur*, including malassezin, activate the aryl hydrocarbon receptor, a transcription factor that regulates various physiological functions of the host, including the immune system. This receptor is also involved in *Malassezia*-related skin disease (Gaitanis George et al. 2008, Lamas et al. 2018). *M. furfur* is also present in healthy individuals (Tajima et al. 2008, Prohic et al. 2014); therefore, identifying the developmental factors of *Malassezia*-related diseases has become more complex.

*Caenorhabditis elegans* is used as an alternative model host for pathogen infection studies owing to its short life cycle and conserved innate immune pathways, such as the mitogen-activated protein kinase (MAPK) and insulin signaling pathways, as well as a wide range of available genetic tools (Finch and Ruvkun 2001, Nicholas and Hodgkin 2004). Pathogenic bacteria and fungi, such as *Salmonella typhimurium* and *Candida albicans*, respectively, shorten the lifespan of *C. elegans* (Labrousse et al. 2000, Pukkila-Worley et al. 2011). In contrast, lactic acid bacteria and *Bifidobacterium* exert probiotic effects that prolong the lifespan of *Salmonella*-infected *C. elegans* (Ikeda et al. 2007). Thus, *C. elegans* is a suitable host model for evaluating the protective effects of probiotics against pathogens.

In this study, we aimed to elucidate the effect of *Malassezia* on the gut and to investigate the mechanisms underlying the innate immune response to it using *C. elegans*. Additionally, we evaluated the effects of lactic acid bacteria on the host’s protection against *M. furfur* pathogenicity.

## Materials and methods

### Bacterial strains and culture conditions

*Malassezia furfur* NBRC0656, *Lacticaseibacillus rhamnosus* (LR) NBRC14710, *Lactobacillus helveticus* (LH) NBRC15019, and *Lactiplantibacillus plantarum* (LP) NBRC15891 were sourced from the Biological Resource Center of the National Institute of Technology and Evaluation (NBRC, Tokyo, Japan). *Malassezia furfur* was cultured in CHROMagar Malassezia/Candida media (Kanto Chemical, Tokyo, Japan) or modified CHROMagar prepared by removing the chromogenic mix and chloramphenicol from standard CHROMagar (38 g/L Bacto Peptone, 2 g/L glycerin, 10 g/L Tween 40, 15 g/L agar) at 30°C for 48-72 h. Unless otherwise specified, Malassezia cultures were performed using CHROMagar medium. Lactic acid bacteria were cultivated anaerobically in No. 804 medium (LR and LH), and in MRS medium (LP; Kanto Chemical). No. 804 medium was prepared by mixing 5.0 g/L Bacto peptone (Thermo Fisher Scientific, Waltham, MA, USA), 5.0 g/L yeast extract (Thermo Fisher Scientific), 5.0 g/L glucose (Fujifilm Wako Pure Chemical, Osaka, Japan), and 1.0 g/L MgSO_4_·7H_2_O (Nacalai Tesque, Kyoto, Japan), and 20 g/L agar (Fujifilm Wako Pure Chemical) was added as needed. *Escherichia coli* OP50 was grown on tryptone soya agar (Shimadzu Diagnostics, Tokyo, Japan) at 37°C for 24 h and used as *C. elegans* standard feed. The bacterial/fungal lawns were suspended in M9 buffer at 10 mg/50 µL to feed *C. elegans*. Heat-killed organisms were prepared by incubation at 100°C for 15 min. The bacterial/fungal suspension (50 μL) was spread on 5-cm peptone-free-modified nematode growth media (mNGM; 1.7% w/v agar, 50 mM NaCl, 1 mM CaCl_2_, 5 μg/mL cholesterol, 25 mM KH_2_PO_4_, and 1 mM MgSO_4_) plates.

### Nematodes and growth conditions

*C. elegans* Bristol strain N2 (wild-type) and its loss-of-function mutant strains AU3 *nsy-1* (*ag3*), KU4 *sek-1* (*km4*), KU25 *pmk-1* (*km25*), CZ4213 *mkk-4* (*ju91*), and VC8 *jnk-1* (*gk7*) were provided by the Caenorhabditis Genetics Center, University of Minnesota (Minneapolis, MN, USA). The nematodes were maintained and propagated on nematode growth media following an established method (Brenner 1974). Eggs were obtained by exposing mature *C. elegans* to a solution containing sodium hypochlorite and sodium hydroxide. The eggs were cultivated in M9 buffer at 25°C for 1 d to promote hatching and synchronization. Synchronized larval stage 1 worms were collected via centrifugation, transferred onto fresh mNGM plates spread with OP, and incubated at 25°C for 2 d until they reached the young adult (3-d-old) stage. To prevent contamination by progeny and dead worms due to internal hatching, 3-d-old *C. elegans* were treated with 2'-deoxy-5-fluorouridine (Tokyo Chemical Industry, Tokyo, Japan) for all experiments (Mitchell et al. 1979).

### Lifespan assay

Synchronized 3-d-old N2 or loss-of-function mutant worms were placed on 5-cm mNGM plates (40 animals per plate) already spread with bacteria and/or fungi and incubated at 25°C. Worms were transferred daily to fresh plates for the first 4 d and every other day thereafter. The number of live and dead worms was recorded daily or every other day. A worm was considered dead if it failed to respond to gentle touch with a worm picker. Worms that crawled into the agar or died by adhering to the plate wall were excluded from the analyses. Worm survival curves were calculated using the Kaplan–Meier method. Mean life span (MLS) and standard error (SE) were estimated described previously (Wu et al. 2006). Data of MLS ± SE for all experiments in this study are provided in Table S1.

### Measurement of body size

Three-day-old adult worms were placed on mNGM plates with bacteria and/or fungi and incubated at 25°C. The size of live worms was measured every 24 h until they reached 6 d of age. Images of mature nematodes were captured and quantified using an all-in-one fluorescence microscope (BZ-X800, Keyence, Osaka, Japan) and a hybrid cell count application (BZ-H3C, Keyence) in the BZ-X Analyzer software (BZ-H3A, Keyence). The worm projection area was used as the body size index. The assay was performed with >40 worms per group for each time point.

### Intestinal barrier function assay (Smurf assay)

The integrity of the intestinal barrier function was examined according to a previously described method (Kim J and Moon 2019), with modifications. Three-day-old adult worms were placed on mNGM plates containing the feed described above and incubated at 25°C. The worms were removed from the mNGM plates at 4–6 d of age or at 6, 10, and 13 d of age and further cultured in liquid media containing HK-OP mixed with blue food dye (Acid Blue 9; Tokyo Chemical Industry, 5% [wt/vol] in NGM liquid solution) at 25°C for 3 h. The worms were washed in M9 buffer until the blue color of the dye was not visible. Then, the worms were observed on a slide with 50 mM sodium azide for the presence or absence of blue food dye in the body cavity using an upright microscope (BX53, Olympus, Tokyo, Japan) and a digital camera (DP73, Olympus) at x100 magnification. The stained worms were counted to determine body cavity leakage without conducting a blind test. Three independent experiments were performed with >15 worms per group for each time point.

### RNA extraction and sequencing

Three-day-old worms were placed on mNGM plates coated with OP or *M. furfur* and incubated at 25°C for 6 h. Approximately 200 worms per group (n = 3) were collected, washed at least three times with M9 buffer containing 0.2% gelatin, and stored in RNAprotect Tissue Reagent (Qiagen, Hilden, Germany) at −80°C until RNA extraction. Then, the thawed nematode suspensions were ground using a microtube pestle (Scientific Specialties, Lodi, CA, USA) and total RNA was extracted using TRIzol reagent (Thermo Fisher Scientific) and the RNeasy Mini Kit (Qiagen). RNA sequencing was performed by DNAFORM (Yokohama, Japan) using 2 out of every 3 samples as previously described (Tsuru et al. 2021). The read count of gene features was performed using the feature Counts tool (version 1.6.1) (Liao et al. 2014), following which quantitative differential expression analysis was conducted between the control (N2_OP) and test-fed groups (N2_*M. furfur*) using DESeq2 (version 1.30.0) (Love MI et al. 2014). Enrichment analysis based on the GO categories for BP, molecular functions, and CC was performed using clusterProfiler (version 3.6.0) (Yu et al. 2012) on genes that exhibited a |log_2_FC| of ≥2 and a base mean of ≥5.

### Reverse transcription and real-time PCR

cDNA was generated using the QuantiTect Reverse Transcription Kit (Qiagen). Real-time quantitative PCR was conducted using the StepOnePlus Real-Time PCR Systems (Thermo Fisher Scientific) with the Power SYBR Green PCR Master Mix (Thermo Fisher Scientific) under the following thermal cycling conditions: initial denaturation at 95°C for 10 min, followed by 40 cycles of denaturation at 95°C for 15 s and annealing/extension at 60°C for 1 min. Samples from three biological replicates were analyzed. Relative mRNA expression was calculated using the ∆∆Ct method (Pfaffl 2001) and normalized to the average expression levels of the three reference genes: *act-1, tba-1*, and *cyc-1*. The primers used for real-time PCR are listed in Table S2.

### Statistical analyses

Statistical analyses were performed using the GraphPad Prism 8 software (GraphPad Prism Software, San Diego, CA, USA). The survival of *C. elegans* was calculated using the Kaplan–Meier method, and statistical significance was assessed using the log-rank (Mantel-Cox) test. Body size, intestinal barrier function, and relative mRNA expression were compared using one-way ANOVA and Tukey’s multiple comparison test.

## Results

### Pathogenicity of *M. furfur* in wild-type (N2) *C. elegans*

**Figure 1:**
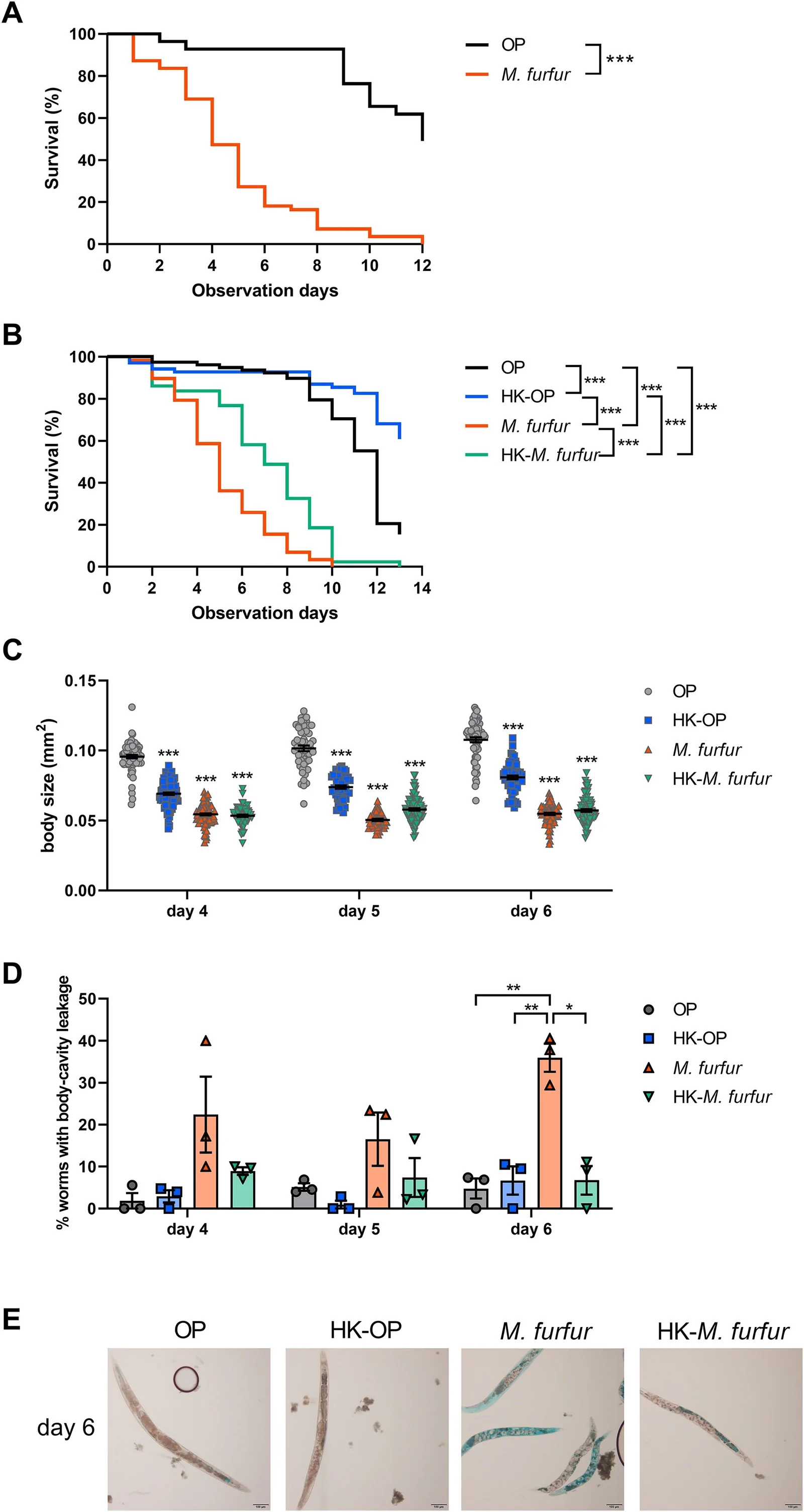
Evaluation of *Malassezia furfur* pathogenicity in *Caenorhabditis elegans*. (A) Survival curves of N2 (wild-type) worms fed *Escherichia coli* OP50 (OP, n = 55) or *M. furfur* (*M. furfur*, n = 55). (B) Survival curves of N2 worms fed live OP or live *M. furfur*, and heat-killed (HK)-OP or HK-*M. furfur* (OP, n = 78; HK-OP, n = 69; *M. furfur*, n = 58; HK-*M. furfur*, n = 43). For (A) and (B), young adult worms (3-d-old) represent day 0 of observation. Survival rates were calculated using the Kaplan–Meier method and asterisks indicate a significant difference compared to OP-fed worms (control) using the log-rank (Mantel-Cox) test. ***, *P* < 0.001. (C) Body size of worms fed OP, HK-OP, *M. furfur*, or HK-*M. furfur* from 4 to 6 d of age. Asterisks indicate significant differences compared to OP-fed worms (control). (D) The intestinal barrier function was assessed by examining the percentage of worms showing leakage of dye into the body-cavity (Smurf assay). Worms fed OP, HK-OP, *M. furfur*, or HK-*M. furfur* were examined from 4 to 6 d of age. For(C) and (D), results are shown as individual plots and means ± standard error of mean (SEM). Statistical analysis was performed with one-way ANOVA and Tukey’s multiple comparison tests. ***, *P* < 0.001; **, *P* < 0.01; *, *P* < 0.05. (E) Representative images of 6-d-old worms fed OP, HK-OP, *M. furfur*, or HK-*M. furfur* and stained with blue dye. Scale bar, 100 µm.

To examine the influence of *M. furfur* infection on the lifespan of *C. elegans*, N2 worms were fed *M. furfur* or *Escherichia coli* OP50 (OP, control). The results showed that N2 worms fed *M. furfur* had significantly shorter lifespans than those fed OP (Fig. 1A). To determine whether live *M. furfur* was pathogenic, lifespan assays were conducted with N2 worms fed heat-killed (HK)-OP or HK-*M. furfur*. It was confirmed that *M. furfur* did not survive after heat treatment at 100°C for 15 min (Fig. S1). The survival rate of nematodes in the HK-*M. furfur* group was significantly lower than that of those in the HK-OP group, whereas the survival rate of worms fed HK-*M. furfur* was higher than that of those fed live *M. furfur* (Fig. 1B). To characterize the pathogenicity of *M. furfur*, we examined the changes in body size and intestinal barrier damage in worms fed *M. furfur*. Worm projection areas were measured beginning at 4 d of age (the day after the start of feeding *M. furfur*) to 6 d of age as an index of body size. Both *M. furfur*- and HK-*M. furfur*-fed groups showed a significant decrease in body area compared to the OP-fed control group (Fig. 1C). Intestinal barrier damage was evaluated using the Smurf assay, in which worms were fed a blue dye and examined for leakage of dye from the intestinal lumen into the body cavity (Laranjeiro et al. 2019). For 6-d-old worms, the percentage of worms with body cavity leakage in the *M. furfur*-fed group was significantly higher than that of those in the other groups. In contrast, no significant differences in intestinal barrier damage were observed among the OP, HK-OP, and HK-*M. furfur* groups (Fig. 1D and E).

### Effect of *M. furfur* infection on MAPK mutants

**Figure 2:**
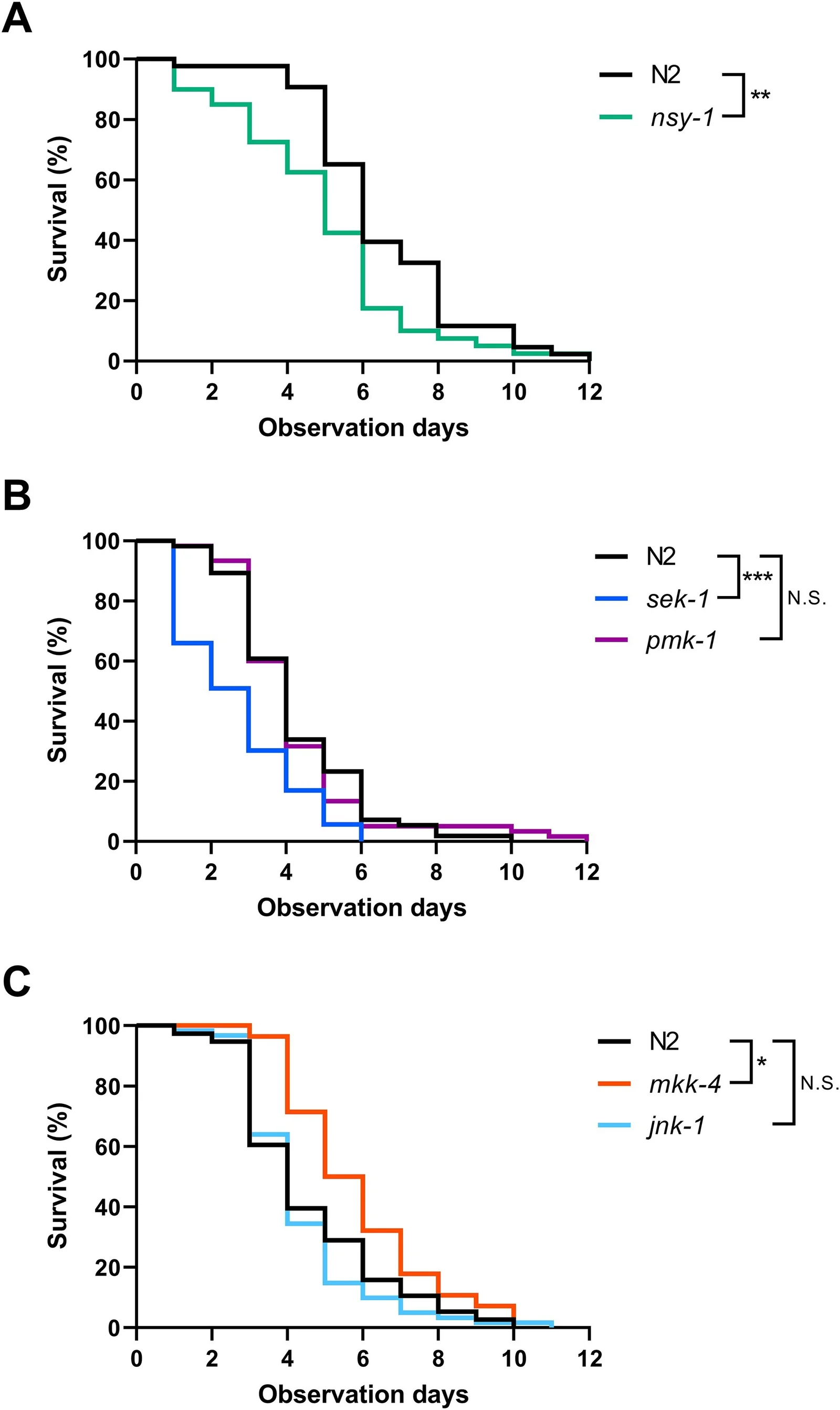
Lifespan assay of *Caenorhabditis elegans* loss-of-function mutants fed *Malassezia furfur*. (A) Survival curves of N2 (wild-type) and *nsy-1* loss-of-function mutant worms fed *M. furfur* (N2, n = 43; *nsy-1*, n = 40). (B) Survival curves of N2 and p38 pathway loss-of-function mutants (*sek-1* and *pmk-1*) worms fed *M. furfur* (N2, n = 56; *sek-1*, n = 53; *pmk-1*, n = 60). (C) Survival curves of N2 and c-Jun N-Terminal Kinase (JNK) pathway loss-of-function mutants (*mkk-4* and *jnk-1*) worms fed *M. furfur* (N2, n = 38; *mkk-4*, n = 28; *jnk-1*, n = 61). For (A) through (C), young adult worms (3-d-old) represent day 0 of observation. Survival rates were calculated using the Kaplan–Meier method and asterisks indicate a significant difference compared to N2 worms (control) using the log-rank (Mantel-Cox) test. ***, *P* < 0.001; **, *P* < 0.01; *, *P* < 0.05; N.S., not significant.

To investigate whether the innate immune response against *M. furfur* in *C. elegans* is mediated by MAPK signal transduction pathways, lifespan assays were performed with *M. furfur*-fed loss-of-function mutants of the *nsy-1, sek-1, pmk-1, mkk-4*, and *jnk-1* genes that encode kinases in the p38 or c-Jun N-Terminal Kinase (JNK) MAPK cascade (Sakaguchi et al. 2004, Wang et al. 2017). The survival rates of *nsy-1* and *sek-1* loss-of-function mutants were significantly lower than those of the N2 (control) worms (Fig. 2A and B, Figs S1 and 2). In contrast, the survival rates of *pmk-1* and *jnk-1* loss-of-function mutants did not differ significantly from the controls, while the survival rate of the *mkk-4* loss-of-function mutant was higher than that of the controls. (Fig. 2B and C, Fig. S2). *pmk-1* encodes p38 MAPK, which is present downstream of the *C. elegans* ortholog of mammalian apoptosis signal-regulating kinase (ASK) family of MAPK kinase kinases (MAPKKK) *nsy-1* and the MAPK kinase (MAPKK) *sek-1*. While the survival rates of the *nsy-1* and *sek-1* mutants were shortened, those of the *pmk-1* mutants were not. We also evaluated the effects of RNAi-mediated *pmk-1* knockdown. The results showed that the survival rate of the *pmk-1* knockdown nematodes was not affected by *M. furfur* infection; this result is similar to that observed for the *pmk-1* mutant (Fig. S3).

### Effect of *M. furfur* infection on *C. elegans* genes

**Figure 3:**
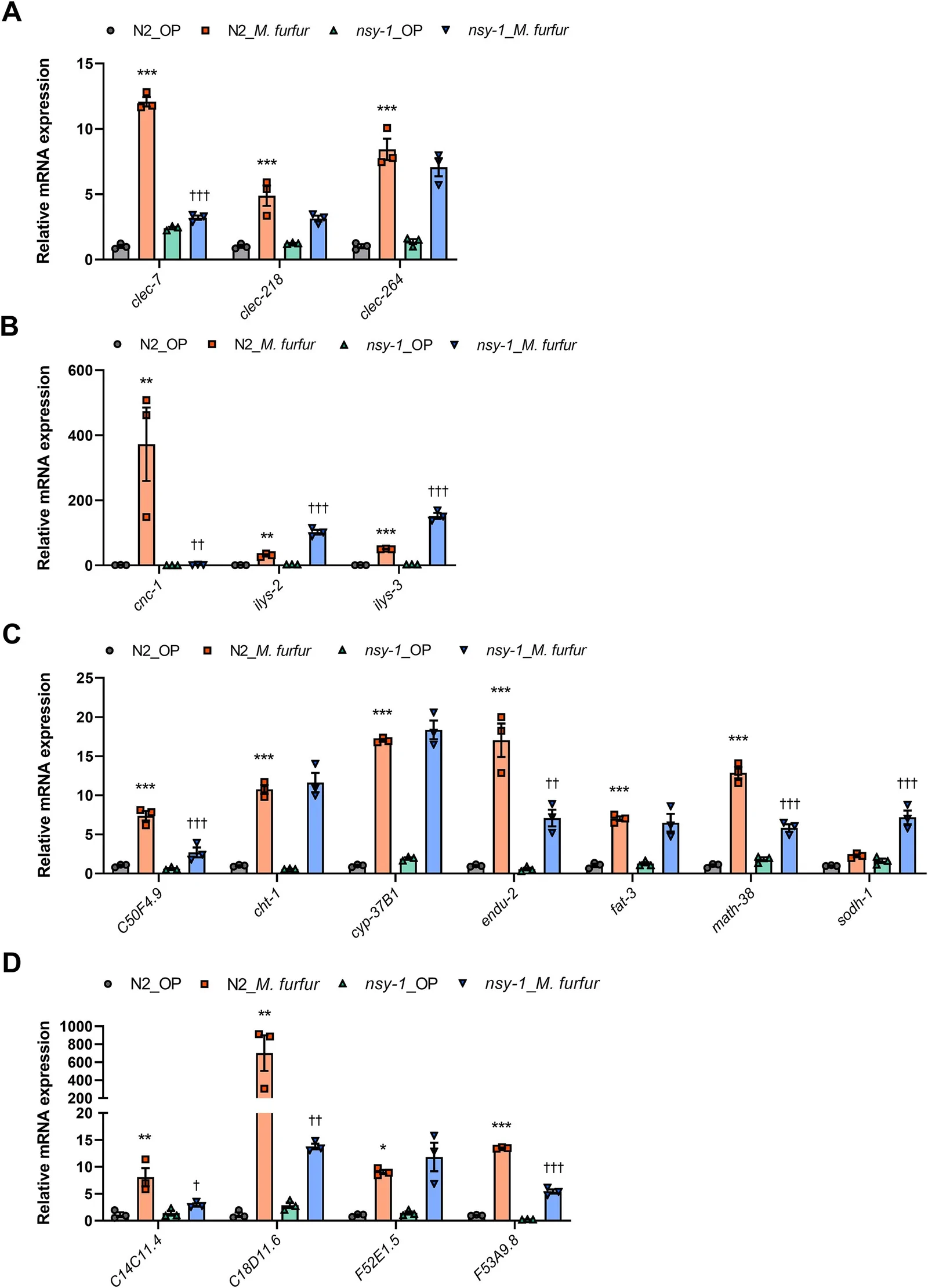
Expression of genes related to top-ranked Gene Ontology terms that were enriched in the N2 (wild-type) *Malassezia furfur*-fed *Caenorhabditis elegans* group. Genes related to C-type lectin domain-containing protein **(A)**, antimicrobial peptide **(B)**, other functions in response to biotic stimulus excluding those for C-type lectin or antimicrobial peptide (C), and unknown detailed function **(D)** are shown. For (A) through (D), real-time PCR was used to determine mRNA expression levels of genes in N2 and *nsy-1* loss-of-function mutants fed *Escherichia coli* OP50 (OP) or *M. furfur* relative to the control group (N2_OP). Data are presented as means ± standard error of mean (SEM) of three independent experiments, each normalized to the average expression levels of the three reference genes (*act-1, cyc-1*, or *tba-1*). Asterisks indicate statistically significant differences between N2_OP and N2_*M. furfur* groups. ***, *P* < 0.001; **, *P* < 0.01; *, *P* < 0.05. Daggers indicate statistically significant differences between N2_*M. furfur* and *nsy-1*_*M. furfur* groups. †††, *P* < 0.001; ††, *P* < 0.01; †, *P* < 0.05.

RNA sequencing was performed to comprehensively investigate the genes, the expression of which was affected by *M. furfur* infection (Table S3). Gene Ontology (GO) enrichment terms were determined for genes upregulated or downregulated by >4-fold following *M. furfur* infection. For genes with elevated expression in the *M. furfur*-fed group, GO terms for biological processes (BP) associated with biological defense, such as “response to biotic stimulus,” were significantly enriched (Table S4). The downregulated genes in the *M. furfur*-fed group were similarly enriched in GO terms associated with biological defense, such as ”immune system process" (Table S5). GO analysis was performed for genes, the expression of which increased in the *M. furfur*-fed N2 group compared to that in the OP-fed N2 group, and mRNA expression levels were quantified using real-time PCR. Of the 22 upregulated genes associated with biological defense, five were excluded because they could not be evaluated using real-time PCR. The PCR results showed significant increase in the expression of three genes encoding C-type lectin domain-containing proteins (Fig. 3A) and three encoding antimicrobial peptides (Fig. 3B) in *M. furfur*-fed N2 group. Additionally, of the seven genes categorized into other functions, six (*C50F4.9, cht-1, cyp-37B1, endu-2, fat-3*, and *math-38*) were significantly upregulated in the *M. furfur*-fed N2 group, whereas one (*sodh-1*) was not (Fig. 3C). Furthermore, four genes (*C14C11.4, C18D11.6, F52E1.5*, and *F53A9.8*) with unknown detailed functions were also upregulated (Fig. 3D).

Real-time PCR was performed to determine whether specific genes in the *nsy-1* loss-of-function mutant are involved in the immune response to *M. furfur*. The results showed that the expression of eight genes (*clec-7, cnc-1, C50F4.9, endu-2, math-38, C14C11.4, C18D11.6*, and *F53A9.8*) was significantly suppressed in the *M. furfur*-fed *nsy-1* loss-of-function mutant group compared to that in the *M. furfur*-fed N2 group. In contrast, the expression of *ilys-2, ilys-3*, and *sodh-1* was significantly increased in the *nsy-1* loss-of-function mutant group. No significant differences were observed in the expression of six genes (*clec-218, clec-264, cht-1, cyp-37B1, fat-3*, and *F52E1.5*) between the *M. furfur*-fed N2 group and *M. furfur*-fed *nsy-1* loss-of-function mutant group (Fig. 3A through D).

### Effect of lactic acid bacteria and *M. furfur* mixture feeding on the lifespan of *C. elegans*

**Figure 4:**
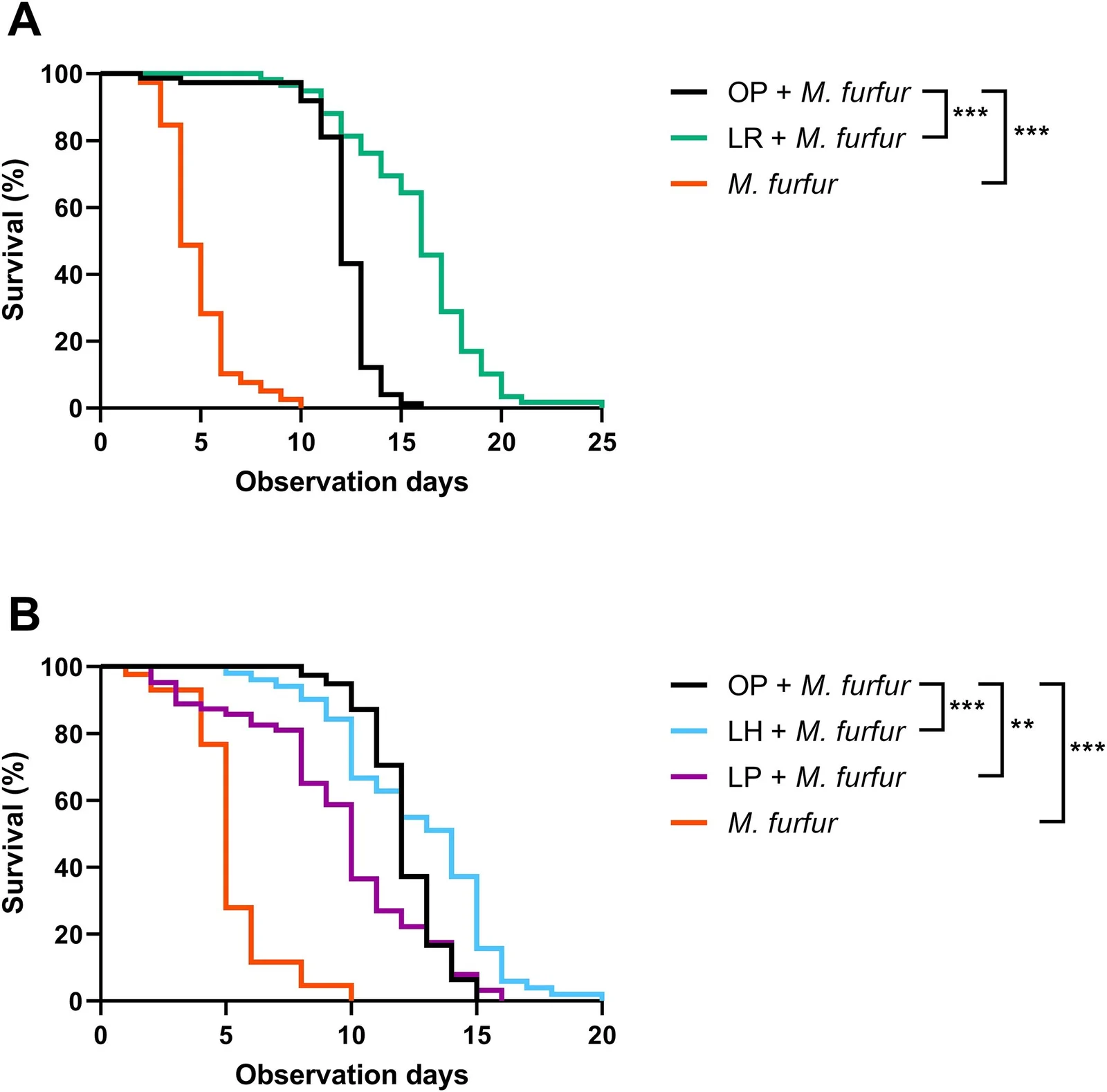
Effects of lactic acid bacteria on the lifespan of *Caenorhabditis elegans* fed *Malassezia furfur*. (A) Survival curves of N2 (wild-type) worms fed with a 1:1 mixture of *Escherichia coli* OP50 and *M. furfur* (OP + *M. furfur* (1:1), n = 74), a 1:1 mixture of *Lacticaseibacillus rhamnosus* and *M. furfur* (LR + *M. furfur* (1:1), n = 59), or only *M. furfur* (*M. furfur*, n = 39). (B) Survival curves of N2 worms fed a 1:1 mixture of OP and *M. furfur* (OP + *M. furfur* (1:1), n = 78), a 1:1 mixture of *Lactobacillus helveticus* and *M. furfur* (LH + *M. furfur* (1:1), n = 51), a 1:1 mixture of *Lactiplantibacillus plantarum* and *M. furfur* (LP + *M. furfur* (1:1), n = 63), or only *M. furfur* (*M. furfur*, n = 43). For (A) and(B), young adult worms (3-d-old) represent day 0 of observation. Survival rates were calculated using the Kaplan–Meier method and asterisks indicate a significant difference compared to OP + *M. furfur*-fed worms, using the log-rank (Mantel–Cox) test. ***, *P* < 0.001; **, *P* < 0.01.

We examined whether feeding lactic acid bacteria, known for their effects on longevity and resistance to *Salmonella* infection in *C. elegans* (Ikeda et al. 2007), could improve the lifespan of worms shortened by *M. furfur* pathogenicity. Lifespan assays showed that N2 worms fed a 1:1 mixture of *Lacticaseibacillus rhamnosus* (LR) or *Lacticaseibacillus helveticus* (LH) and *M. furfur* had significantly longer lifespans than those fed OP + *M. furfur* (Fig. 4A and B). In contrast, N2 worms fed a 1:1 mixture of *Lactiplantibacillus plantarum* (LP) and *M. furfur* did not show improved survival rates (Fig. 4B). We also examined the intestinal lumen of *C. elegans* fed a mixture of LR and *M. furfur*. In the worms in the LR + *M. furfur* group, both LR and *M. furfur* were detected in the intestines (Fig. S4).

### Effect of *L. rhamnosus* on *M. furfur* pathogenicity

**Figure 5:**
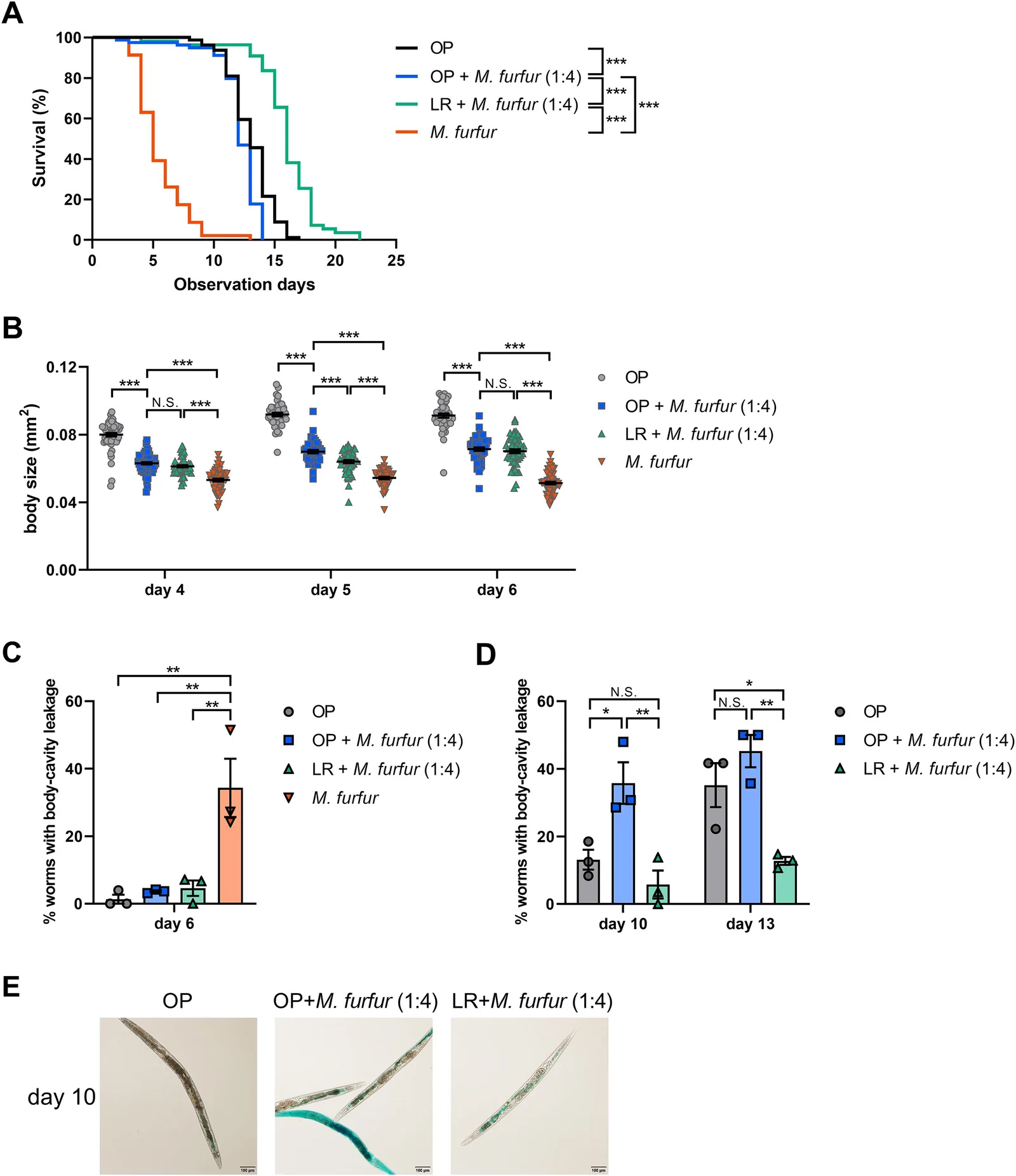
Evaluation of lactic acid bacteria protection against *Malassezia furfur* virulence in *Caenorhabditis elegans*. (A) Survival curves of N2 worms fed a mixture with a high proportion of *M. furfur* (OP, n = 79; OP + M. furfur (1:4), n = 79; LR + *M. furfur* (1:4), n = 55; *M. furfur*, n = 46). Young adult worms (3-d-old) represent day 0 of observation. Survival rates were calculated using the Kaplan–Meier method and asterisks indicate a significant difference compared to OP + *M. furfur*-fed worms using the log-rank (Mantel–Cox) test. ***, *P* < 0.001; *, *P* < 0.05. (B) Body size of worms fed OP, OP + *M. furfur* (1:4), LR + *M. furfur* (1:4), or only *M. furfur* from 4 to 6 d of age. Asterisks indicate significant differences compared to OP + *M. furfur* (1:4)-fed worms. (C) The percentage of body-cavity leakage worms fed OP, OP + *M. furfur* (1:4), or LR + *M. furfur* (1:4) was compared to those fed *M. furfur* at 6 d of age. (D) The percentage of worms with body-cavity leakage fed OP and LR + *M. furfur* (1:4) was compared to those fed OP + *M. furfur* (1:4) at 10 and 13 d of age. For (B) through (D), results are shown as individual plots and means ± standard error of mean (SEM). Statistical analysis was performed using one-way ANOVA and Tukey’s multiple comparison tests. ***, *P* < 0.001; **, *P* < 0.01; *, *P* < 0.05; N.S., not significant. **(E)** Representative images of 10-d-old worms fed OP, OP + *M. furfur* (1:4), or LR + *M. furfur* (1:4) and stained with blue dye. Scale bar, 100 µm.

Among the tested lactic acid bacteria, LR showed the greatest improvement in the survival rate of worms infected with *M. furfur*; therefore, the lifespan assay was conducted with an increased proportion of *M. furfur* (LR + *M. furfur* (1:4)). Even with a higher proportion of *M. furfur*, the survival rate of worms in the LR + *M. furfur* (1:4) group was better than that of those in the OP + *M. furfur* (1:4) group (Fig. 5A). To evaluate the effect of LR on *M. furfur* pathogenicity, changes in body size and intestinal barrier damage in worms fed a 1:4 mixture of LR and *M. furfur* were assessed. Projection areas were measured in worms beginning at 4 d of age (the day after grouping) to 6 d of age. The body area of worms did not differ significantly between the OP + *M. furfur* and LR + *M. furfur* groups (Fig. 5B). The Smurf assay was performed on worms of age 6, 10, and 13 d. At 6 d of age, nematodes in the *M. furfur* group showed more body cavity leakage than those in the other groups, while no significant differences were observed among the OP, OP + *M. furfur*, and LR + *M. furfur* groups (Fig. 5C). At 10 d of age, nematodes in the LR + *M. furfur* group showed significantly lower body-cavity leakage than those in the OP + *M. furfur* group. Additionally, at 13 d of age, worms in the LR + *M. furfur* group displayed fewer dye leaks into the body cavity than those in the OP and OP + *M. furfur* groups (Fig. 5D and E).

## Discussion

This study was conducted to characterize *M. furfur* pathogenicity and evaluate the protective effects of probiotics, specifically lactic acid bacteria, against it, using *C. elegans* as a model host. We first assessed the impact of *M. furfur* infection on nematodes to evaluate its virulence. *M. furfur*-fed *C. elegans* exhibited significantly shorter lifespans than the controls, indicating that *M. furfur* is pathogenic to nematodes. Additionally, HK-*M. furfur* also shortened the lifespan of nematodes, although to a lesser extent than live *M. furfur* did. Heat-killed *Cryptococcus neoformans*, the yeast that causes cryptococcosis, is pathogenic to nematodes, suggesting that part of the pathogenicity of this yeast may be due to cell wall fragments or toxins (Mylonakis et al. 2002). In the present study, compared to OP-feeding, both *M. furfur* and HK-*M. furfur* infection significantly reduced the body surface area of *C. elegans. Malassezia* contains pathogenic factors, such as galactomannans in the cell wall, and various indole compounds, including malassezin (a tryptophan metabolite) (Krämer et al. 2005, Gaitanis George et al. 2008, Shibata et al. 2009). Aryl hydrocarbons are heat-stable; therefore, part of *M. furfur*’s pathogenicity could be attributed to cellular components (CC), such as metabolites and cell wall of the fungus, which are less affected by heating. Furthermore, *C. albicans* mutants that have a reduced ability to form mycelia are significantly less toxic to nematodes (Pukkila-Worley et al. 2009). Similar to *C. albicans, M. furfur* can exist in both yeast and hyphal forms (Sudbery et al. 2004, Gaitanis Georgios et al. 2013); therefore, mycelial formation may also play a significant role in the pathogenicity of *M. furfur*. The increased survival observed in the HK-OP group may be attributable to reduced bacterial pathogenicity and/or a dietary restriction-like state resulting from reduced food intake or nutritional quality of heat-killed bacteria (Thériault et al. 2025). Conversely, the reduced body size may reflect the low nutritional quality of heat-killed bacteria, including the loss of heat-sensitive micronutrients such as vitamins (Qi et al. 2017). In the present study, several nematodes fed live *M. furfur* exhibited dye leakage, suggesting that *M. furfur* damages the intestinal barrier. *C. elegans* lacks acquired immunity; therefore, the intestinal barrier is an important defense mechanism against pathogenic microorganisms. A similar disruption is caused by enteropathogenic *E. coli* in *C. elegans* (Kim J and Moon 2019). In contrast, HK-*M. furfur* did not cause significant intestinal damage, suggesting that virulence factors of dead *M. furfur* do not include components responsible for intestinal destruction. Paraformaldehyde treatment is known as an effective method for inactivating OP50 (Thériault et al. 2025). Therefore, using paraformaldehyde instead of heat treatment for *M. furfur* inactivation may result in different effects on *C. elegans* phenotypes. Further studies comparing different inactivation methods are needed to determine the influence of *M. furfur* inactivation methods of on the host.

*M. furfur-*fed *nsy-1* and *sek-1* loss-of-function mutant *C. elegans* showed a decrease in lifespan, while the *pmk-1* and *jnk-1* loss-of-function mutants showed no change in lifespan. Notably, as compared to that of N2, the lifespan of the *mkk-4* loss-of-function mutant was extended after *M. furfur* infection, suggesting that this mutant may be detrimental to *M. furfur*. NSY-1 is a homolog of the mammalian MAPKKK ASK1 that activates JNK and p38 MAPKs (33), and the p38 MAP kinase pathway consists of NSY-1-SEK-1-PMK-1 (Sagasti et al. 2001). Mammalian MKK4 MAPKK activates both p38 and JNK MAPKs (34), and MKK-4 and JNK-1 in *C. elegans* encode homologs of mammalian MKK4 and JNK, respectively (Kim DH et al. 2002). These results suggest that *nsy-1* and *sek-1* play important roles in the p38 MAPK-related response to *M. furfur* pathogenicity. In contrast, the knockdown of *pmk-1*, which is thought to function downstream of *nsy-1* and *sek-1*, did not affect the lifespan of *C. elegans*, suggesting that alternative active pathways downstream of *sek-1* may exist. Insulin-like signaling pathways, transforming growth factor-β (TGF-β)-like pathways, and programmed cell death pathways are involved in the response to various pathogen infections in *C. elegans*; these pathways may interact with the MAPK pathway (Aballay A. and Ausubel 2001, Mallo et al. 2002, Aballay Alejandro et al. 2003, Garsin et al. 2003, Kondo et al. 2005, Zugasti and Ewbank 2009).

GO analysis revealed that among the genes, the expression of which increased or decreased more than 4-fold following *M. furfur* infection, a substantial number were involved in defense responses, suggesting that the expression of genes involved in defense responses is regulated by *M. furfur* infection. Further GO analysis revealed that the genes, the expression of which were upregulated by *M. furfur* infection, were involved in the defense response to gram-positive bacteria. This finding suggests that defense responses mechanisms against gram-positive bacteria and fungi may be partially similar in *C. elegans*. In contrast, *M. furfur* infection downregulated the expression of genes involved in the immune system; however, we only focused on genes upregulated by *M. furfur* infection. Real-time PCR of N2 nematodes demonstrated that the expression of *clec-7, clec-218*, and *clec-264*, which encode C-type lectin domain-containing proteins involved in pathogen recognition, along with *cnc-1, ilys-2*, and *ilys-3*, which encode antimicrobial peptides, increased with *M. furfur* infection. C-type lectin-like proteins are involved not only in recognition but also in pathogen elimination as soluble secretory proteins (Schulenburg et al. 2004). Some caenacin genes, including *cnc-1* of *C. elegans*, are induced by infection with the fungus *Drechmeria coniospora* (Zugasti and Ewbank 2009); furthermore, the invertebrate lysozyme *ilys-3* is upregulated upon infection with *C. albicans* (Gravato-Nobre et al. 2016). These results suggest that the expression of multiple genes associated with biological defense is activated in *M. furfur* infection. Additionally, *cht-1*, one of the genes, the expression of which was upregulated in *M. furfur* infection, encodes an endochitinase and is involved in *C. albicans* infection (Pukkila-Worley et al. 2011). In contrast, *sodh-1*, which encodes an alcohol dehydrogenase and has been implicated in the fungus *Purpureocillium lavendulum* infection (Zhuang et al. 2023), did not show increased expression upon *M. furfur* infection. To further investigate the mechanism of the immune response, we compared the gene expression levels in the *nsy-1* loss-of-function mutant *C. elegans* with those in N2 nematodes. Of the 16 genes that showed significant upregulated expression upon *M. furfur* infection in N2, the expression of eight was significantly suppressed in the *nsy-1* loss-of-function mutant nematodes exposed to *M. furfur*, suggesting the dependence of these genes on NSY-1 for their activation in response to *M. furfur*. In contrast, *ilys-2, ilys-3*, and *sodh-1* were upregulated in the *nsy-1* loss-of-function mutant. The expression of *ilys-3* is upregulated in an extracellular signal-regulated MAP kinase (ERK)-MAPK pathway-dependent manner (Gravato-Nobre et al. 2016). The loss of *nsy-1* may have triggered the activation of other immune response pathways to compensate, leading to increased expression of *ilys -2, ilys-3*, and *sodh-1* downstream of these pathways.

*Malassezia* is associated with inflammatory bowel disease (Limon et al. 2019). In the present study, *M. furfur* caused intestinal barrier disruption in *C. elegans*; therefore, we investigated the protective effect of probiotic bacteria in *M. furfur* infection. The results of mixed feeding with each of the three lactic acid bacterial species (LR, LH, or LP) and *M. furfur* showed that the survival rates of the nematodes were higher in the LR or LH and *M. furfur* mixed feeding group than the OP + *M. furfur* group, suggesting that LR and LH protect nematodes from the pathogenic effects of *M. furfur*. The OP + *M. furfur* mixed feeding group also showed an increased survival rate compared to the *M. furfur* infection group, suggesting that OP may also mitigate *M. furfur* virulence, though to a lesser extent than LR. In contrast, the LP + *M. furfur* group did not extend the survival rate of the OP + *M. furfur* group, indicating that different types of lactic acid bacteria offer varying degrees of protection against *M. furfur* pathogenicity. Feeding with LR, LH, or LP extends the lifespan of nematodes compared to feeding with OP (Ikeda et al. 2007), suggesting that lifespan extension and protection against pathogens may be mediated through different mechanisms. For instance, wild-type nematodes pretreated with the isolate *Lacticaseibacillus zeae* before infection with enterotoxigenic *Escherichia coli* (ETEC) showed a significantly extended lifespan compared to those pretreated with OP before infection (Zhou et al. 2018).

Among the three species of lactic acid bacteria, LR provided the most significant improvement in *C. elegans* survival against *M. furfur*; therefore, we evaluated whether feeding LR could alleviate the reduction in nematode body area and intestinal barrier damage caused by *M. furfur*. No significant differences in body area were observed between the OP + *M. furfur* and LR + *M. furfur* groups on the last day of body area measurements, suggesting that although mixed LR feeding did slightly slow the growth rate of nematodes compared to mixed OP feeding, it did not significantly alter the final body size. *Lactobacillus* retention and intestinal distention were also observed in *C. elegans* fed LR + *M. furfur*, indicating that LR does not eliminate the intestinal distention caused by *M. furfur. Enterococcus faecalis* causes intestinal distention and shortens survival, while *E. faecium* causes intestinal distention without affecting the lifespan of *C. elegans* (Yuen and Ausubel 2018), suggesting that intestinal distention and the accumulation of bacteria do not necessarily affect the lifespan of *C. elegans*. It is possible that LR prevented *M. furfur* from contacting the host intestinal tract by colonizing the intestinal tract, thereby preventing the induction of an excessive immune response. Intestinal staining results showed that significantly more 6-d-old nematodes in the *M. furfur* group exhibited dye leakage than those in the OP, OP + *M. furfur*, and LR + *M. furfur* groups, suggesting that mixed feeding with OP or LR could prevent damage to the nematode intestinal barrier caused by *M. furfur*. Moreover, significantly fewer 10- and 13-d-old nematodes in the LR + *M. furfur* group showed dye leakage than those in the OP + *M. furfur* group, suggesting that LR protects the intestinal barrier more effectively than OP. *Lacticaseibacillus zeae* regulates nematode signaling and the production of antimicrobial peptides and other defense effectors during ETEC infection (Zhou et al. 2018). The LR strain Lcr35 significantly inhibited *C. albicans* growth and adhesion to Caco-2 cells in vitro (Poupet et al. 2019). The protective mechanism of LR in *M. furfur* infection in nematodes may include direct antimicrobial effects against *M. furfur* via antimicrobial peptides, and/or indirect effects via activation of host immunity, such as strengthening the intestinal barrier and enhancing intestinal repair.

This study had some limitations. In this study, FUdR was used to prevent contamination from the progeny or dead nematodes resulting from internal hatching; therefore, the use of FUdR may have affected the lifespan of the nematodes. Furthermore, as the study examined only one species of the genus Malassezia—*M. furfur*—and only one strain of LR, it cannot be guaranteed that the findings will be consistent across all strains of the genera *Malassezia* and LR. The mechanism by which LR protects against *M. furfur* is yet to be elucidated. Functional validation of the C-type lectin encoding “*clec*” and antimicrobial peptide (AMP) gene clusters identified as candidates in RNA-Seq was not performed, and it is unclear how these genes are related to *M. furfur* infections. Although *pmk-1* was not involved in *M. furfur* infection in this study, the role of *nsy-1*/*sek-1* in other pathways remains unclear.

**Figure 6:**
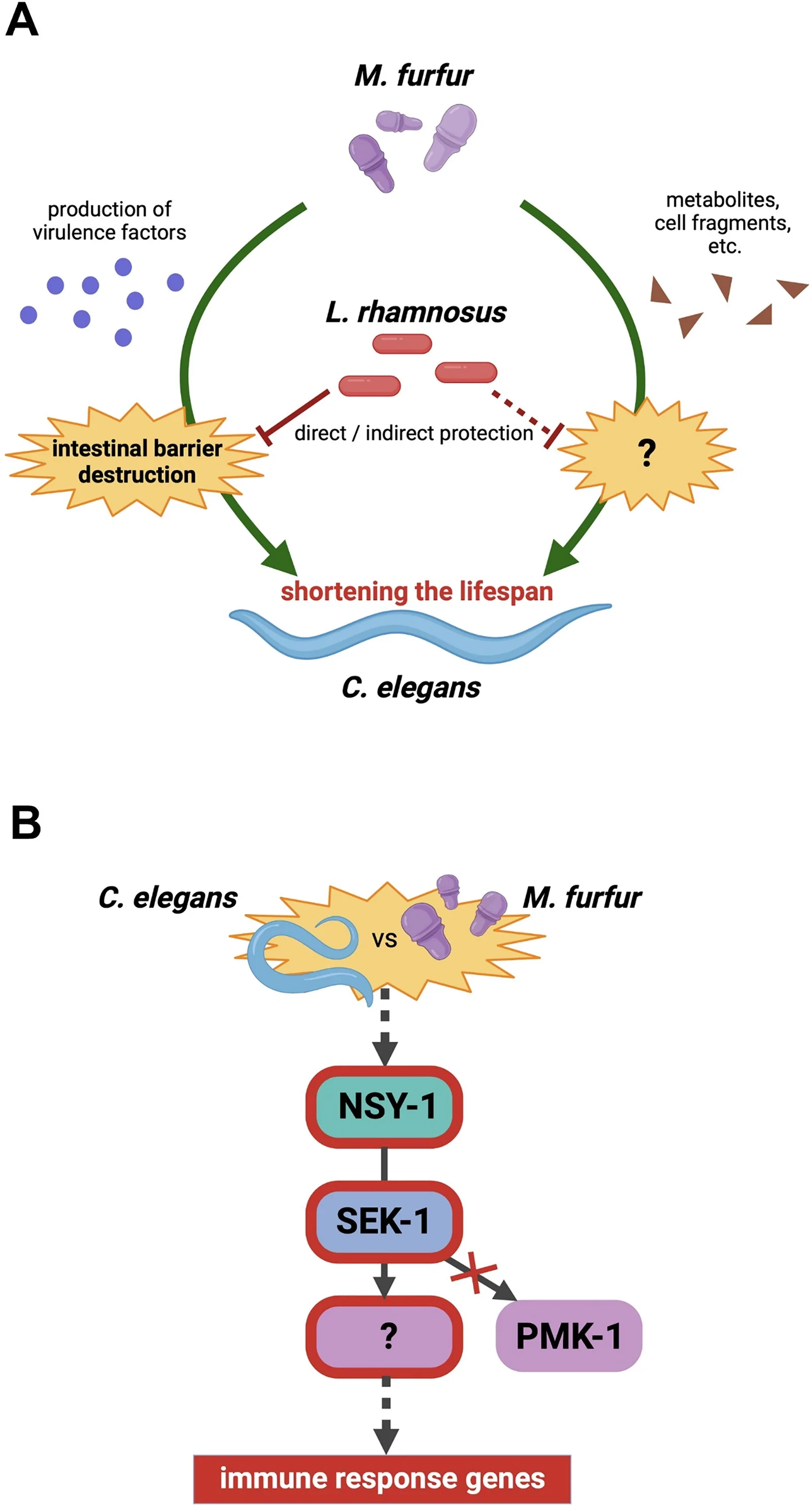
Schematic representation of the pathogenicity of *Malassezia furfur* in *Caenorhabditis elegans*.(A)The protective effect of *Lacticaseibacillus rhamnosus*. (B) Hypothetical model of the signaling pathway that acts in *C. elegans* upon *M. furfur* infection.

In conclusion, this study characterized the virulence of *M. furfur* and identified the protective effect of LR against *M. furfur* infection in *C. elegans* (Fig. 6A). The shortened lifespan of *C. elegans* infected with *M. furfur* was linked to the p38 signaling pathways specifically involving *nsy-1* and *sek-1* (Fig. 6B). Although *pmk-1* has been reported to be a factor that acts downstream of *sek-1* (Sagasti et al. 2001), *pmk-1* was not involved in *M. furfur* infection, suggesting that there may be another unknown pathway. Gene expression analyses in *C. elegans* exposed to *M. furfur* showed elevated expression levels for genes involved in host defense, C-type lectin domain-containing proteins, and antimicrobial peptides. We believe that our findings will lead to a better understanding of the diseases associated with *M. furfur* and provide insights into developing novel preventive and therapeutic approaches using probiotics.

## Supplementary Material

xtag049_Supplemental_Files

## Data Availability

Data supporting the findings of this study are available from the Gene Expression Omnibus (GEO) (GSE260976).
