# Supplementary material for: Pathogenicity and intestinal barrier disruptive ability of Malassezia furfur in an alternative model host Caenorhabditis elegans is partially alleviated by Lacticaseibacillus rhamnosus
Source: FEMS Microbes. 2026 Sep 8;7:xtag049. doi: 10.1093/femsmc/xtag049 (PMC13587822; doi:10.1093/femsmc/xtag049)
Supplement: Supplementary file 1 [file xtag049_supplemental_files.zip › FEMSMC-2026-031.R1 supplementary data - reviews and author response.docx]

**Reviews of manuscript FEMSMC-2026-031.R1**

**Reviewer 1**

**Recommendation: Reject**

The majority of the data presented in this paper comes from a single biological replicate, and the number of biological replicates performed for each type of experiment is not explicitly stated in the methods, except for the Smurf assay. The lack of experimental rigor must be addressed for publication.

Major Issues

1) Figs 1A, 1B, 2A, 2B, 2C, 4, 4B, 5A, 5B: Lifespan assays must be performed in biological triplicate, and all data from replicates must be presented.

2) Fig 3: Which reference gene was used for normalization in each panel? Three reference genes are listed in the materials and methods.

3) Transcriptomics: How many biological replicates were submitted to RNA seq? Why genes with an adjusted p-value > 0.05 are included in the differentially expressed gene list (Table S3)

**Reviewer 2**

**Recommendation: Minor Revision**

Overview

The manuscript by Kishida and colleagues establishes C. elegans as a practical model host for investigating Malassezia furfur pathogenicity, which appears to be a research gap (or new opportunity) to learn more about mechanisms beyond current research in the context of skin biology/infection. Key results demonstrate that both live and heat-killed M. furfur shorten wild-type N2 lifespan and reduce body size compared to control OP50 E. coli. Also, live cells cause significantly greater lifespan reduction and intestinal barrier disruption (via Smurf assay with blue dye leakage).

The authors go further using genetic analysis of innate immune response genes. This includes the p38 MAPK pathway and genes nsy-1, sek-1, pmk-1 and jnk-1. They show that some genes modify phenotypes (nsy-1, sek-1, mkk-4) but not others (pmk-1, jnk-1), shedding light on genetic requirements for M. furfur toxicity. The authors do a good job of employing transcriptional regulation studies (RNA-seq followed by RT-PCR) to better understand genes regulated in response to M. furfur.

The last section involves co-culturing M. furfur with bacterial strains, namely probiotic Lacticaseibacillus rhamnosus strains where they observed rescue of M. furfur-induced toxicity phenotypes (lifespan, body size and intestinal leakage).

The discussion is thoughtful and goes on to propose direct (competition, perhaps by preventing MF from contacting the intestinal tract) and indirect (immune modulation) mechanisms to explain Lacticaseibacillus rhamnosus protective activity.

Strengths

Generally well written and logical. The abstract seems a little terse and could be improved

The work is novel and relevant. The use of C. elegans for a lipid-dependent fungus like Malassezia is innovative and fills a methodological gap for studying gut/skin microbe-host interactions, with clear translational links to several pathological conditions.

Good use of multiple whole-organism approaches, generally well done (with good numbers and statistical analyses).

The inclusion of HK-M. furfur distinguishes live virulence from cell wall/metabolite effects. Multiple lactic acid bacteria were tested for specificity; FUDR was used to control progeny.

The graphical abstract is helpful. The use of supplementary tables is good, and having a summary table for lifespan data (Table S1) is appreciated at this stage of the submission process.

I feel like the probiotic rescue angle adds practical value for FEMS Microbes readership.

Weaknesses

The abstract seems a little terse and could be improved. Maybe introduce what Malassezia furfur is right away; not everyone knows it is a fungus, and thus the manuscript’s novelty is increased since the work looks at fungus/bacteria interactions, and specifically probiotics vs. fungus/pathogens. Also, just listing gene names (nsy-1, sek-1) in the abstract doesn't convey much information. Some additional info about what they are would aid the reader.

FUDR is used to control progeny. There is a trend to get away from this approach given the effects it can have on experiments. Perhaps list this as a limitation.

Limited Strain Diversity. Only one M. furfur strain was tested, so results may not generalize across the 18-species/clinical isolates. Similarly, probiotic effects are strongest for LR but variable (LP ineffective). Also, any information on which strain of Lacticaseibacillus rhamnosus was used? There are a lot of them in use these days.

While nsy-1/sek-1 involvement is shown, the lack of pmk-1 effect is noted but not deeply explored (alternative pathways hypothesized but untested).

Gene expression changes are correlative, but functional validation (e.g., RNAi of key clec/AMPs?) is absent.

How LR protects (antifungal metabolites? physical exclusion? immune priming?) remains speculative.

Heat-killing validation (viability check post 100°C/15 min) is not explicitly described.

Culture conditions for M. furfur (CHROMagar) and lipid supplementation in C. elegans plates could be clarified, as Malassezia is lipid-dependent (at least to my understanding).

Smurf assay timing and replicates are good, but could specify blinding.

Figure 1. Interesting that survival was greater with HK-OP, but body size was smaller. Any ideas why?

Maybe an alternative to heat-killing? Paraformaldehyde inactivation seems to be a robust approach (PubMed ID: 41276563).

**Associate Editor Recommendation: Major Revision**

Thank you for submitting this manuscript to FEMS Microbes. The reviewers recognized several strengths of the study, including the use of C. elegans as an alternative host model for Malassezia furfur, the analysis of intestinal barrier disruption, and the potential protective effects of Lacticaseibacillus rhamnosus. I agree that the manuscript addresses an interesting and potentially useful topic for the journal.

However, the manuscript is not yet suitable for acceptance in its current form. The primary concern is that the biological replication supporting several central conclusions is unclear or appears insufficient. In particular, the lifespan and body-size assays form a major foundation of the study, and the revised manuscript should clearly state the number of independent biological replicates for each experiment and present the replicate-level data where appropriate. If these experiments have not been performed with adequate independent biological replication, they should be repeated.

The transcriptomic analysis also requires clarification. Please state how many biological replicates were used for RNA-seq, clarify the criteria used to define differentially expressed genes, and ensure that the deposited data include the raw sequencing data or provide the relevant accession information.

Several additional issues raised by the reviewers should also be addressed, including clarification of heat-killing validation, culture conditions for M. furfur, normalization of RT-qPCR data, limitations associated with the use of FUDR and a single M. furfur strain, and appropriate tempering of mechanistic conclusions regarding probiotic protection.

I am therefore inviting a major revision. A revised manuscript should include a detailed point-by-point response to both reviewers and should clearly indicate how the concerns regarding experimental replication, RNA-seq reporting, and interpretation of the data have been addressed. A substantially revised manuscript will be considered further, but acceptance cannot be guaranteed at this stage.

**EIC Decision: Major Revision**

**Author's Response to Decision Letter for (FEMSMC-2026-031)**

Response to comments

Reviewer: 1

The majority of the data presented in this paper comes from a single biological replicate, and the number of biological replicates performed for each type of experiment is not explicitly stated in the methods, except for the Smurf assay. The lack of experimental rigor must be addressed for publication.

[Reply] We thank the Reviewer for the valuable comments. We have provided point-by-point responses to your comments below. The revised text in the manuscript is highlighted in yellow.

Major Issues

1) Figs 1A, 1B, 2A, 2B, 2C, 4, 4B, 5A, 5B: Lifespan assays must be performed in biological triplicate, and all data from replicates must be presented.

[Reply] We reviewed our past data and repeated experiments for the three experiments that were not replicated. Since Fig. 4A and 5A depicted the same experiment, Fig. 5A was moved to the supplementary figures. These results are summarized in the supplementary figures and tables.

2) Fig 3: Which reference gene was used for normalization in each panel? Three reference genes are listed in the materials and methods.

[Reply] Relative mRNA expression levels were normalized relative to the average expression levels of the three reference genes: act-1, tba-1, and cyc-1. We have amended the Materials and Methods section.

3) Transcriptomics: How many biological replicates were submitted to RNA seq? Why genes with an adjusted p-value > 0.05 are included in the differentially expressed gene list (Table S3)

[Reply] We extracted RNA from three biological replicates and performed RNA-seq using 2 of every 3 samples. We used only metrics with a |log2FC| of ≥2 and base mean of ≥5 to narrow down the genes, and we did not take p-values into account. Table S3 includes genes with p-values >0.05. We have added this information to the revised Materials and Methods section for improved clarity.

Reviewer: 2

Overview

The manuscript by Kishida and colleagues establishes C. elegans as a practical model host for investigating Malassezia furfur pathogenicity, which appears to be a research gap (or new opportunity) to learn more about mechanisms beyond current research in the context of skin biology/infection. Key results demonstrate that both live and heat-killed M. furfur shorten wild-type N2 lifespan and reduce body size compared to control OP50 E. coli. Also, live cells cause significantly greater lifespan reduction and intestinal barrier disruption (via Smurf assay with blue dye leakage).

The authors go further using genetic analysis of innate immune response genes. This includes the p38 MAPK pathway and genes nsy-1, sek-1, pmk-1 and jnk-1. They show that some genes modify phenotypes (nsy-1, sek-1, mkk-4) but not others (pmk-1, jnk-1), shedding light on genetic requirements for M. furfur toxicity. The authors do a good job of employing transcriptional regulation studies (RNA-seq followed by RT-PCR) to better understand genes regulated in response to M. furfur.

The last section involves co-culturing M. furfur with bacterial strains, namely probiotic Lacticaseibacillus rhamnosus strains where they observed rescue of M. furfur-induced toxicity phenotypes (lifespan, body size and intestinal leakage).

The discussion is thoughtful and goes on to propose direct (competition, perhaps by preventing MF from contacting the intestinal tract) and indirect (immune modulation) mechanisms to explain Lacticaseibacillus rhamnosus protective activity.

Strengths

Generally well written and logical. The abstract seems a little terse and could be improved

The work is novel and relevant. The use of C. elegans for a lipid-dependent fungus like Malassezia is innovative and fills a methodological gap for studying gut/skin microbe-host interactions, with clear translational links to several pathological conditions.

Good use of multiple whole-organism approaches, generally well done (with good numbers and statistical analyses).

The inclusion of HK-M. furfur distinguishes live virulence from cell wall/metabolite effects. Multiple lactic acid bacteria were tested for specificity; FUDR was used to control progeny.

The graphical abstract is helpful. The use of supplementary tables is good, and having a summary table for lifespan data (Table S1) is appreciated at this stage of the submission process.

I feel like the probiotic rescue angle adds practical value for FEMS Microbes readership.

[Reply] We thank the Reviewer for their valuable comments. We have provided point-by-point responses to your comments below. The revised text in the manuscript is highlighted in yellow.

Weaknesses

The abstract seems a little terse and could be improved. Maybe introduce what Malassezia furfur is right away; not everyone knows it is a fungus, and thus the manuscript’s novelty is increased since the work looks at fungus/bacteria interactions, and specifically probiotics vs. fungus/pathogens. Also, just listing gene names (nsy-1, sek-1) in the abstract doesn't convey much information. Some additional info about what they are would aid the reader.

[Reply] Thank you for your comment. As per your suggestion, we have added explanations regarding M. furfur, relationship between probiotics and fungi, and the nsy-1 and sek-1 genes to the abstract.

FUDR is used to control progeny. There is a trend to get away from this approach given the effects it can have on experiments. Perhaps list this as a limitation.

[Reply] Thank you for your comment. We have added a note regarding the potential impacts of using FUdR as a limitation in the Discussion section.

Limited Strain Diversity. Only one M. furfur strain was tested, so results may not generalize across the 18-species/clinical isolates. Similarly, probiotic effects are strongest for LR but variable (LP ineffective). Also, any information on which strain of Lacticaseibacillus rhamnosus was used? There are a lot of them in use these days.

[Reply] As you pointed out, we have only examined one species of the Malassezia genus, M. furfur, and only one strain of LR. We have noted this as a limitation. We used the LR strain NBRC 14710. We have already described this in the “Result” section as follows: “We examined whether feeding lactic acid bacteria, known for their effects on longevity and resistance to Salmonella infection in C. elegans (22), …”

While nsy-1/sek-1 involvement is shown, the lack of pmk-1 effect is noted but not deeply explored (alternative pathways hypothesized but untested).

[Reply] In this study, we could only demonstrate that pmk-1 is not involved in M. furfur infection. We have noted this as a limitation.

Gene expression changes are correlative, but functional validation (e.g., RNAi of key clec/AMPs?) is absent.

[Reply] As you pointed out, we could not conduct functional validation of these genes. We have noted this as a limitation.

How LR protects (antifungal metabolites? physical exclusion? immune priming?) remains speculative.

[Reply] The mechanism underlying the protective effect of LR against M. furfur infection has not yet been elucidated. We have noted this as a limitation.

Heat-killing validation (viability check post 100°C/15 min) is not explicitly described.

[Reply] We added a supplementary figure showing that M. furfur did not survive heat treatment at 100°C for 15 minutes and we have also included this information in the Results section.

Culture conditions for M. furfur (CHROMagar) and lipid supplementation in C. elegans plates could be clarified, as Malassezia is lipid-dependent (at least to my understanding).

[Reply] CHROMagar contains glycerin and Tween 40 as lipid sources. Additionally, in these additional experiments, we cultured M. fufur using modified CHROMagar, which was prepared by removing the chromogenic mix and chloramphenicol from CHROMagar. Details regarding these experiments are included in the revised Materials and Methods section. The NGM plates used for nematode culture contain cholesterol, but it is unclear whether M. furfur can grow in this medium. However, as its pathogenicity was reduced when M. furfur was killed, we believe that it was being used as a food source while still alive.

Smurf assay timing and replicates are good, but could specify blinding.

[Reply] We did not conduct the Smurf assay evaluation in a blinded manner. We have added this information to the Materials and Methods section.

Figure 1. Interesting that survival was greater with HK-OP, but body size was smaller. Any ideas why?

[Reply] There are several other reports indicating that heat treatment extends the survival of OP. OP is known to possess mild pathogenicity. However, heat treatment leads to a deficiency in nutrients such as vitamins, and this could be the reason for the reduced body length. We have added this information to Discussion section.

Maybe an alternative to heat-killing? Paraformaldehyde inactivation seems to be a robust approach (PubMed ID: 41276563).

[Reply] We thank the reviewer for pointing this out. Several methods have been reported for bacterial inactivation, including heat treatment and paraformaldehyde treatment. Although paraformaldehyde inactivation may offer an alternative approach, we did not directly compare different inactivation methods in the present study. Therefore, we have noted the lack of comparison with other bacterial inactivation methods in Discussion section.

**Reviews of manuscript FEMSMC-2026-031.R1**

**Reviewer 1**

**Recommendation: Accept**

The authors addressed my concerns from my previous review.

**Reviewer 2**

**Recommendation: Accept**

The authors have adequately addressed my concerns and are clear about the limitations of the study.

**Associate Editor Recommendation: Accept**

I am pleased to inform you that your revised manuscript, “Pathogenicity and intestinal barrier disruptive ability of Malassezia furfur in an alternative model host Caenorhabditis elegans is partially alleviated by Lacticaseibacillus rhamnosus,” has been favorably reviewed; the reviewers and I are satisfied that you have addressed the concerns raised and strengthened the manuscript appropriately. Congratulations, and thank you for choosing FEMS Microbes for your work.

**EIC Decision: Accept**
