# Supplementary material for: Pathogenicity and intestinal barrier disruptive ability of Malassezia furfur in an alternative model host Caenorhabditis elegans is partially alleviated by Lacticaseibacillus rhamnosus
Source: FEMS Microbes. 2026 Sep 8;7:xtag049. doi: 10.1093/femsmc/xtag049 (PMC13587822; doi:10.1093/femsmc/xtag049)
Supplement: Supplementary file 1 [file xtag049_supplemental_files.zip › Kishida_Supplementary_information_R1_20260817.pdf]

Supplementary Information

Material and Methods

Gene knockdown by RNAi treatment

RNA interference (RNAi) was carried out by feeding animals dsRNA-producing bacteria (Ahringer *C. elegans* RNAi feeding library). Synchronized L1-stage worms were transferred to plates containing RNAi bacteria grown on NGM containing 100 µg/mL ampicillin and 1 mM isopropyl-beta-D-thiogalactopyranoside (IPTG), and the plates were incubated for three days at 20 °C until the transferred animals developed into young adults. These animals were used in the lifespan assay.

Microscopic observation

The *L. rhamnosus* cell pellets were harvested from agar plate and incubated for 30 min in 1 mL of phosphate-buffered saline (PBS) containing 0.1 mg of fluorescein isothiocyanate (FITC; Dojindo, Kumamoto, Japan). The *M. furfur* cell pellets were harvested from agar plate, washed three times with PBS, and incubated for 5 min in 0.5 mL of Calcofluor White Stain (CFW; Sigma-Aldrich, St. Louis, MO, USA). The stained cells were washed three times with M9 buffer, resuspended in M9 buffer. Young adult nematodes were placed on NGM plates spread with a 1:1 mixture of FITC-labeled *L. rhamnosus* and CFW-labeled *M. furfur* for 1.5 hours. Images of nematodes were captured using BZ-9000 microscope (Keyence, Tokyo, Japan).

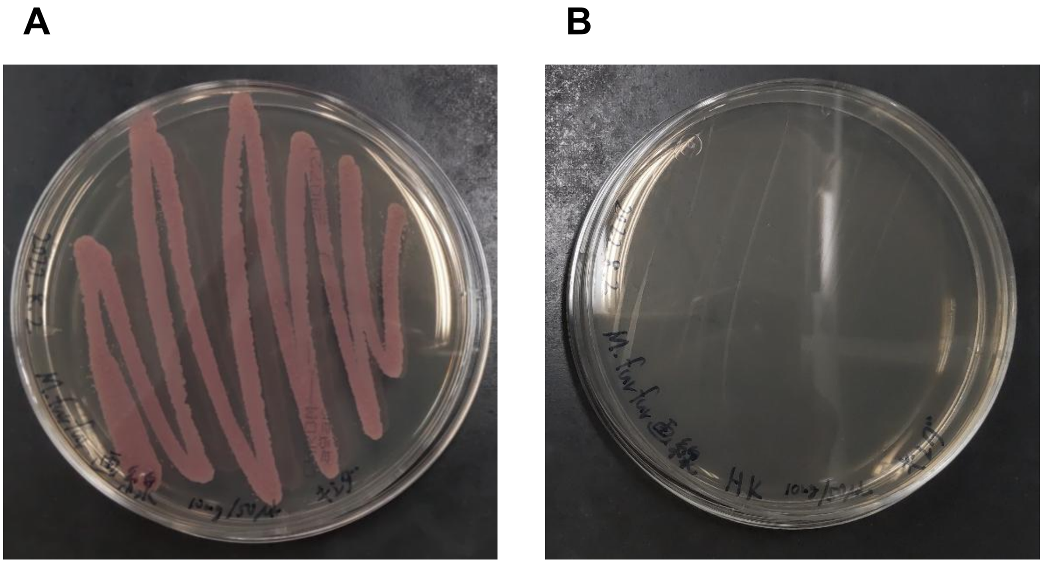

Supplemental Figure S1. Confirmation of the growth of heat-killed *M. furfur*. The *M. furfur* was treated at 100 °C for 15 min and then streaked onto CHROM agar. (A) untreated, (B) heat-treated.

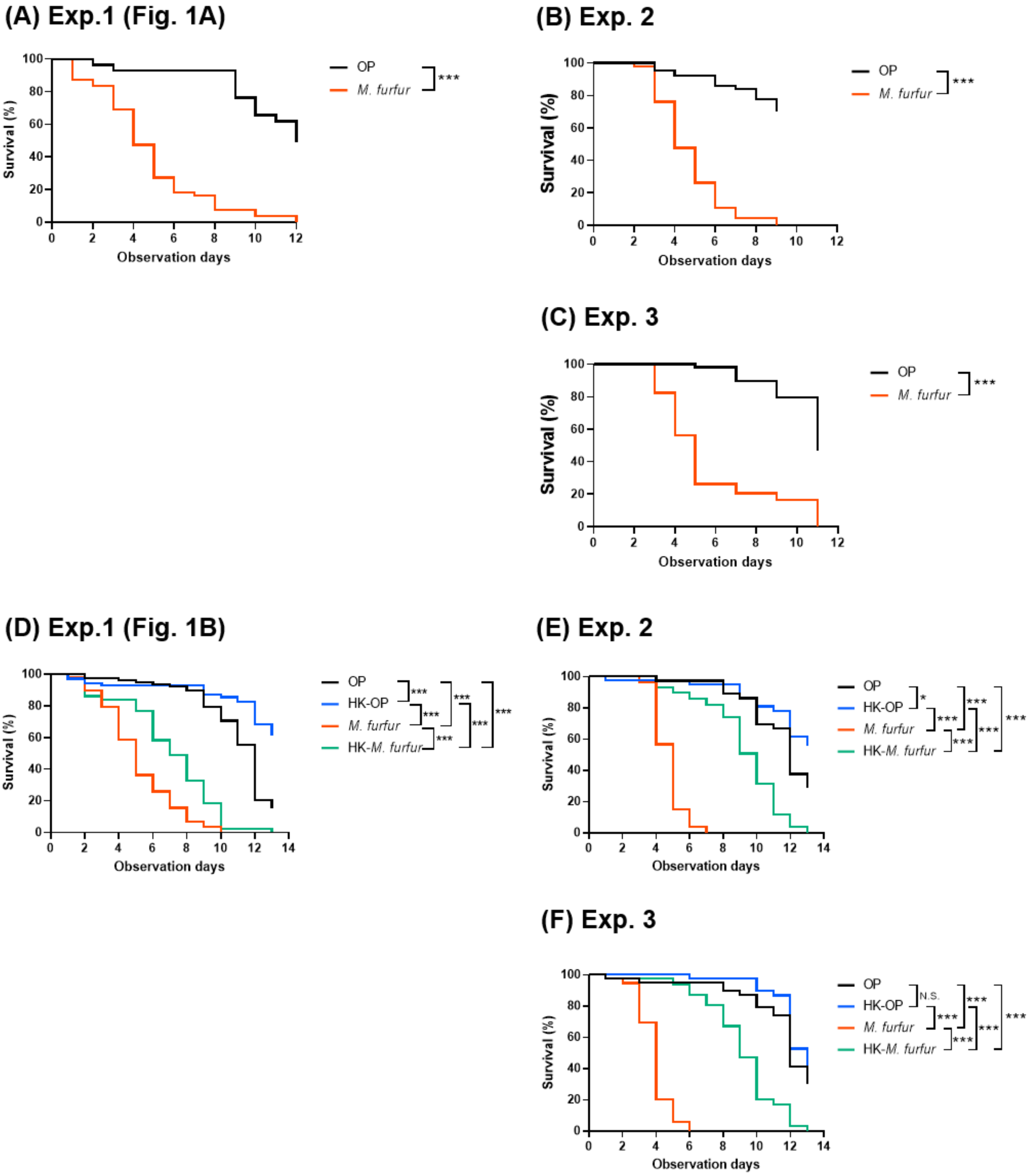

Supplemental Figure S2. Three biologically independent survival assays shown in Figure 1. In (C), (E), and (F), *M. furfur* was cultured using modified CHROM agar.

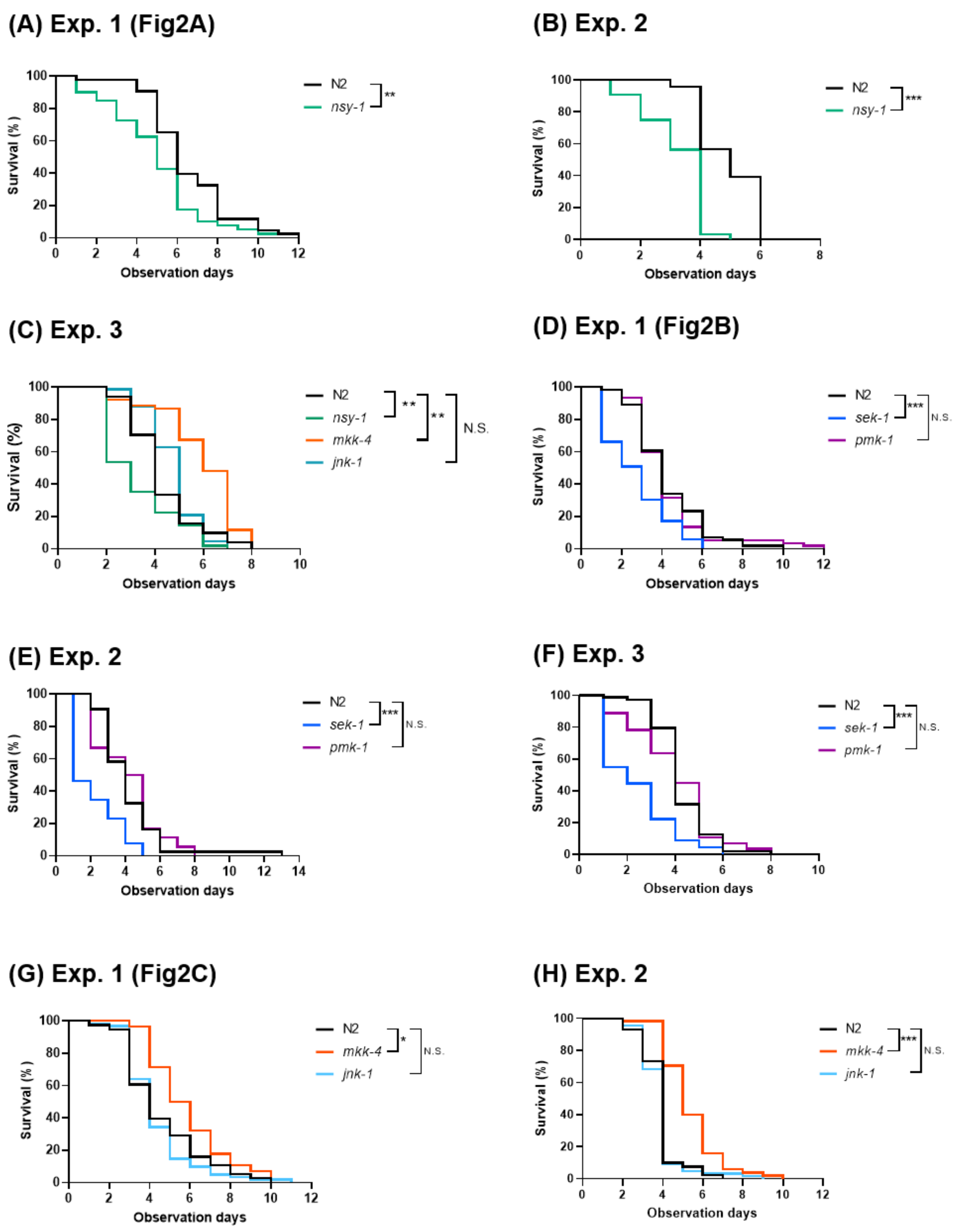

Supplemental Figure S3. Three biologically independent survival assays shown in Figure 2. In (F) and (H), *M. furfur* was cultured using modified CHROM agar.

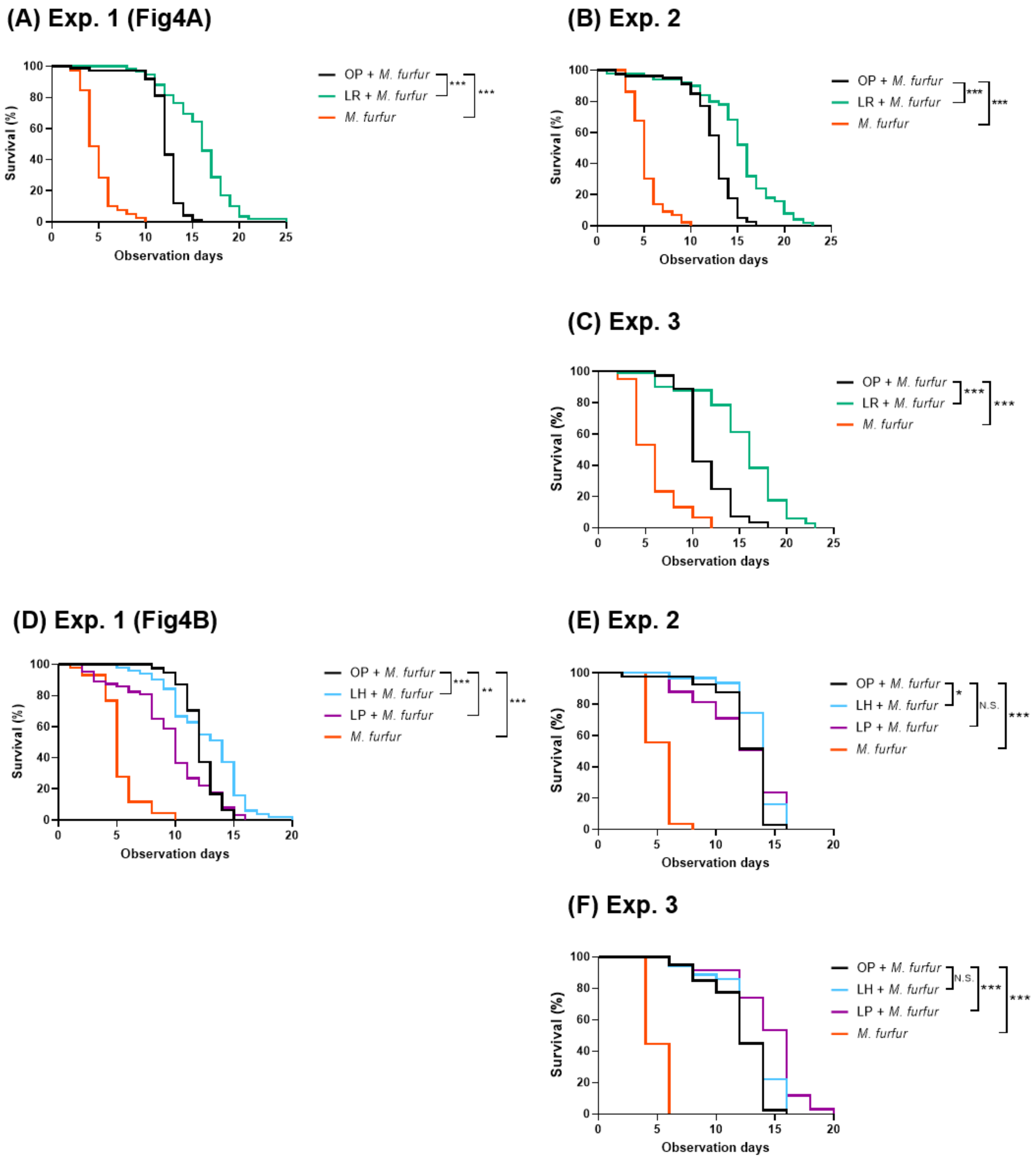

Supplemental Figure S4. Three biologically independent survival assays shown in Figure 4. In (C), (E), and (F), *M. furfur* was cultured using modified CHROM agar.

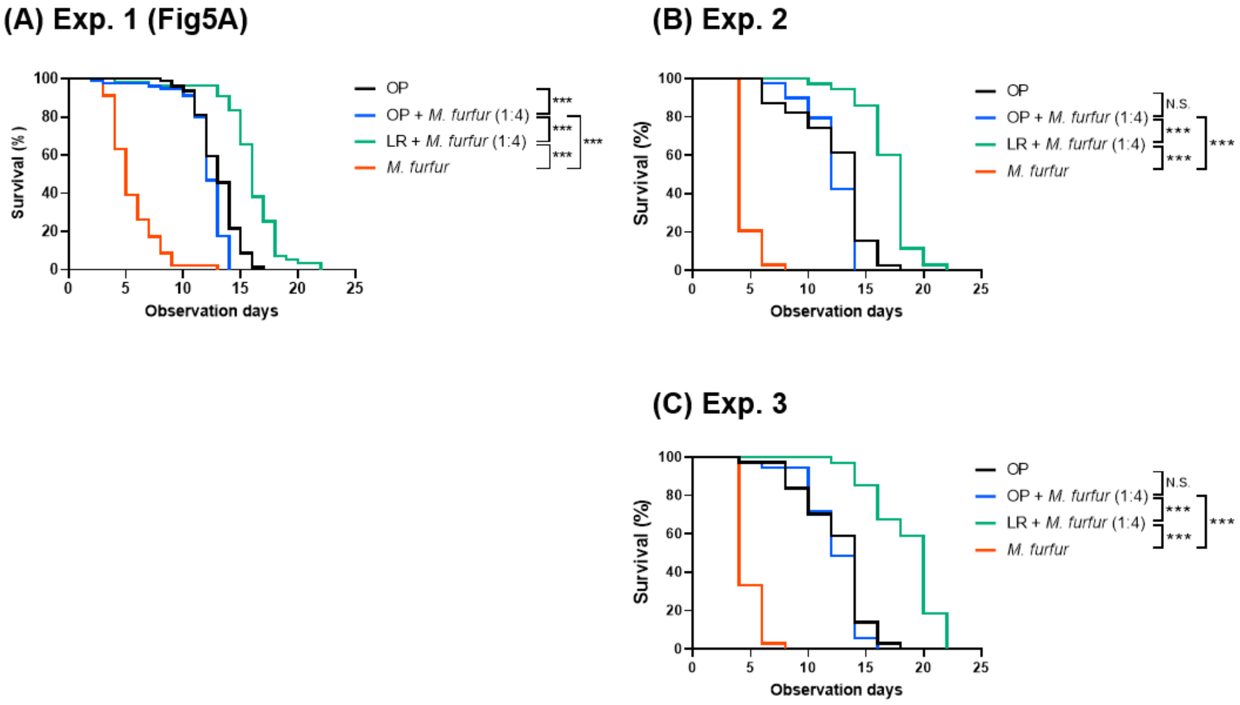

Supplemental Figure S5. Three biologically independent survival assays shown in Figure 5. In (B) and (C), *M. furfur* was cultured using modified CHROM agar.

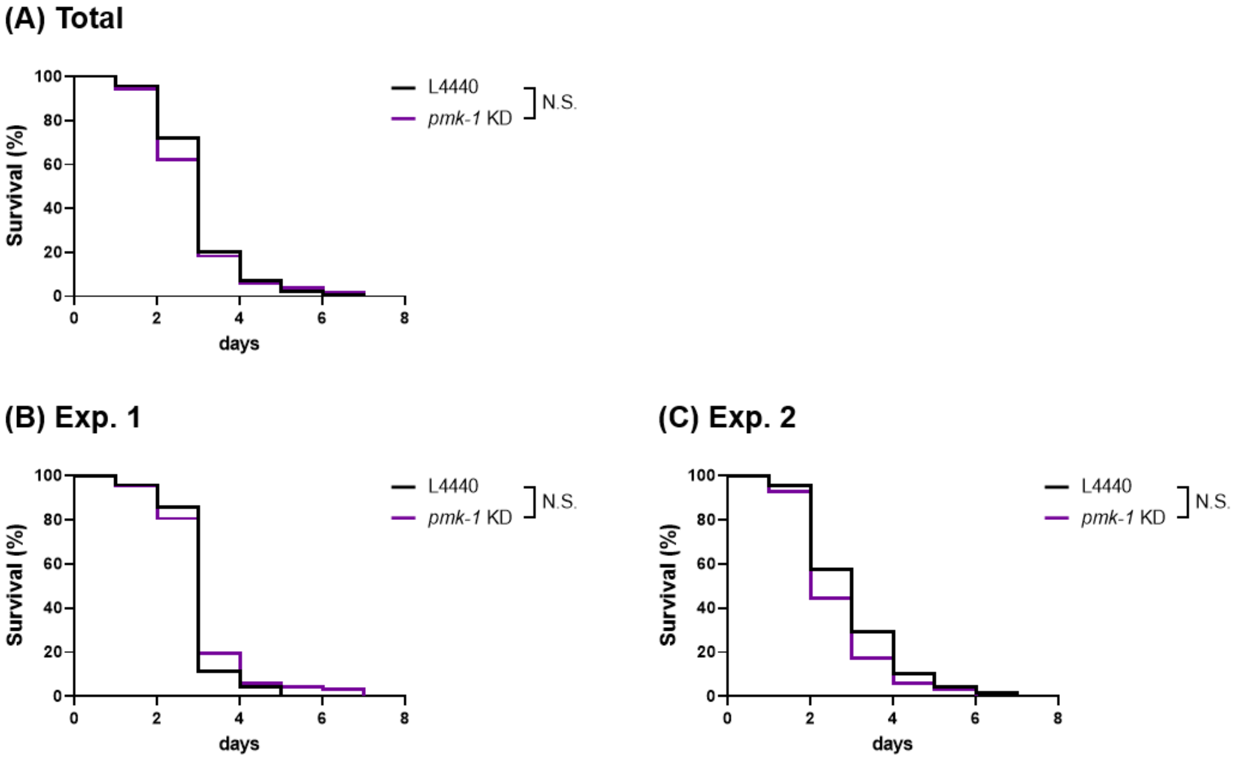

Supplemental Figure S6. Lifespan assays in N2 treated by *pmk-1* RNAi fed with *M. furfur*. Survival curves are shown for the combined results of two experiments (A), the first experiment (B), and second experiment (C). *M. furfur* was cultured using modified CHROM agar.

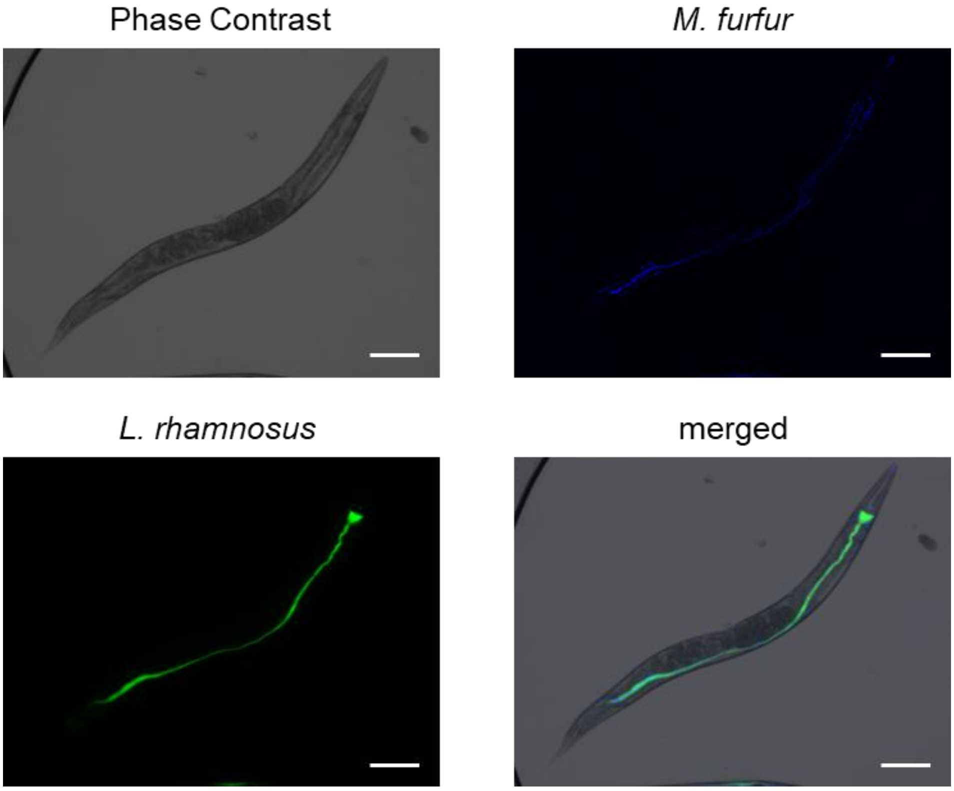

Supplemental Figure S7. Representative intestinal lumen images of worms fed LR + *M. furfur*. Blue indicates *M. furfur*, and green indicates LR. Scale bars, 100 µm.
